# Guidelines proposal for clinical recognition of mouth breathing children

**DOI:** 10.1590/2176-9451.20.4.039-044.oar

**Published:** 2015

**Authors:** Maria Christina Thomé Pacheco, Camila Ferreira Casagrande, Lícia Pacheco Teixeira, Nathalia Silveira Finck, Maria Teresa Martins de Araújo

**Affiliations:** 1 Full professor, Universidade Federal do Espírito Santo, Department of Clinical Dentistry, Vitória, Espírito Santo, Brazil; 2 Private practice, Vitória, Espírito Santo, Brazil; 3 Volunteer professor, Universidade Federal do Espírito Santo, Vitória, Espírito Santo, Brazil; 4 MSc in Clinical Dentistry, Universidade Federal do Espírito Santo, Vitória, Espírito Santo, Brazil; 5 Adjunct professor, Universidade Federal do Espírito Santo, Department of Physiological Sciences, Vitória, Espírito Santo, Brazil

**Keywords:** Mouth breathing, Airway obstruction, Craniofacial abnormalities

## Abstract

**INTRODUCTION:**

Mouth breathing (MB) is an etiological factor for sleep-disordered breathing (SDB) during childhood. The habit of breathing through the mouth may be perpetuated even after airway clearance. Both habit and obstruction may cause facial muscle imbalance and craniofacial changes.

**OBJECTIVE:**

The aim of this paper is to propose and test guidelines for clinical recognition of MB and some predisposing factors for SDB in children.

**METHODS:**

Semi-structured interviews were conducted with 110 orthodontists regarding their procedures for clinical evaluation of MB and their knowledge about SDB during childhood. Thereafter, based on their answers, guidelines were developed and tested in 687 children aged between 6 and 12 years old and attending elementary schools.

**RESULTS:**

There was no standardization for clinical recognition of MB among orthodontists. The most common procedures performed were inefficient to recognize differences between MB by habit or obstruction.

**CONCLUSIONS:**

The guidelines proposed herein facilitate clinical recognition of MB, help clinicians to differentiate between habit and obstruction, suggest the most appropriate treatment for each case, and avoid maintenance of mouth breathing patterns during adulthood.

## Abstract (pt)

**INTRODUÇÃO:**

a respiração bucal (RB) é um fator etiológico para os distúrbios respiratórios do sono (DRS) na infância. O hábito de respirar pela boca pode ser perpetuado mesmo depois da desobstrução das vias aéreas. Tanto o hábito quanto a obstrução podem causar desequilíbrios da musculatura facial e alterações craniofaciais. O objetivo deste trabalho é propor e testar uma diretriz para o reconhecimento clínico da RB e de alguns fatores predisponentes aos DRS em crianças.

**MÉTODOS:**

entrevistas semiestruturadas foram realizadas com 110 ortodontistas, com relação aos seus procedimentos para avaliação clínica da RB e aos seus conhecimentos sobre DRS na infância. A partir daí, com base nas respostas obtidas, uma diretriz foi desenvolvida e testada em 687 crianças, com 6 a 12 anos, oriundas de escolas de ensino fundamental.

**RESULTADOS:**

não existe padronização para o reconhecimento clínico da RB pelos ortodontistas. Os procedimentos mais comumente realizados foram ineficientes para reconhecer a diferença entre a RB por hábito e a por obstrução.

**CONCLUSÕES:**

a diretriz proposta facilita o reconhecimento clínico da RB, diferencia entre RB por hábito e por obstrução, sugere o tratamento mais adequado para cada caso, e evita a manutenção do padrão de respiração bucal na idade adulta.

## INTRODUCTION

Due to its range of comorbidities, mouth breathing (MB) has been a concern for healthcare professionals in various areas.^1^^-^ 4 The most common cause of MB is the presence of obstacles in the nasopharyngeal region,which increases nasal resistance that can be induced by various mechanical factors, including tonsil hyperplasia, hypertrophied turbinates, rhinitis, tumors, infectious or inflammatory diseases, and changes in nasal architecture.^2^^,^ 5 However, even after these mechanical factors are removed, MB continues in most cases due to patient's mouth breathing habit.^4^^,^ 6 Unbalanced facial musculature occurs as a result of MB, which causes changes in tooth positioning, lips, tongue, palate, and jaws, so as to counterbalance the new breathing pattern.^7^^-^ 10

MB is one of the most commonly cited characteristics of sleep-disordered breathing (SDB) during childhood, but symptoms are often inadequately recognized. SDB encompasses a wide clinical spectrum, such as snoring, upper airway resistance syndrome (UARS), and obstructive sleep apnea (OSA).^11^^,^ 12 Snoring during sleep is estimated to occur among 8% and 27% of children, 2% of which present with OSA.^13^^,^ 14 Prevalence of UARS remains unknown and is most likely to be underdiagnosed. Findings for clinical diagnosis of UARS are considered nonspecific, but strongly resemble clinical aspects of chronic mouth breathing and nasal obstruction.^15^^,^ 16 ^,^ 17

Dentists may be the first healthcare professionals to have contact with a MB child. Due to the importance of early detection and the need for appropriate treatment, the present study aimed to investigate the perception of MB by orthodontists, propose guidelines for its clinical recognition, and test the applicability of these guidelines among children aged 6-12.

## MATERIAL AND METHODS

This prospective cross-sectional study was approved by Universidade Federal do Espírito Santo Institutional Review Board under protocol #162/09. All participants signed an informed consent form before data collection. All procedures were performed by trained and calibrated researchers.

The study was carried out with two distinct populations: orthodontists and children. A sample of 110 orthodontists answered a semi-structured questionnaire about clinical evaluation of respiratory patterns during childhood and their knowledge about SDB in children. Data collection was tabulated and analyzed. Lack of standardization of the procedures employed by orthodontists as well as of diagnostic information in the literature led us to prepare basic guidelines to clinically recognize MB in children (Table 1), based on the most cited procedures.

**Table 1.** Proposed guidelines for clinical recognition of mouth breathing

CLINICAL RECOGNITION OF MOUTH BREATHING	
These guidelines can be used to examine children and aid recognition of mouth breathing	
1. Visual assessment	
The dentist should assess at least the presence of the following characteristics:	
With the patient standing:	
» Lack of lip seal	( ) YES ( ) NO
» Posture changes	( ) YES ( ) NO
» Dark eye circles	( ) YES ( ) NO
» Long face	( ) YES ( ) NO
With the patient sited:	
» Anterior open bite	( ) YES ( ) NO
» High narrow palate	( ) YES ( ) NO
» Gingivitis in maxillary incisors	( ) YES ( ) NO
2. Questions	
Questions should be directed to the child or parents	
Do you:	
» Sleep with your mouth open?	( ) YES ( ) NO
» Keep your mouth open when you are distracted?	( ) YES ( ) NO
» Snore?	( ) YES ( ) NO
» Drool on your pillow?	( ) YES ( ) NO
» Experience excessive daytime sleepiness?	( ) YES ( ) NO
» Wake up with a headache?	( ) YES ( ) NO
» Get tired easily?	( ) YES ( ) NO
» Often have allergies?	( ) YES ( ) NO
» Often have a stuffy nose and/or runny nose?	( ) YES ( ) NO
» Have difficulty in school?	( ) YES ( ) NO
» Have difficulty concentrating?	( ) YES ( ) NO
3. Breathing tests	
The child must be sitting. At least two tests should be performed.	
a. Graded mirror test	
After the second output of air on the mirror, mark the halo area with a marker (Fig 1).	
(Low nasal flow: up to 30 mm; Average nasal flow: 30-60 mm; High nasal flow: above 60 mm)	
b. Water retention test	
Place water in the patient’s mouth (approximately 15 ml) and ask him/her to hold it for 3 minutes.	
c. Lip seal test	
Seal the patient’s mouth completely with a tape for 3 minutes.	
4. Training to eliminate the habit of mouth breathing	
Training should be performed at home on a daily basis until the child is able to return to nasal breathing.	
Lip seal test	
Seal the child’s mouth with masking tape when he/she is distracted or focusing his/her attention on another activity. Progressively increase the time each day until the child is able to breathe only through the nose for, at least, two consecutive hours.	

Guidelines presented in Table 1 were applied to 687 children aged 6-12 years old and attending elementary schools. Only healthy children whose parents gave permission to participate were included.

Children were clinically assessed and received diagnostic impressions as mouth breathers or nose breathers according to their clinical characteristics. Subsequently, they were subjected to three breathing tests selected to assist MB recognition: the mirror test, the water retention test and the lip seal test. All tests were performed with the child sitting with his/her head straight, keeping his/her lips closed, and breathing normally.

## RESULTS

The procedures most commonly used by orthodontists for clinical diagnosis of a child's breathing pattern were: patient's visual assessment (97.2%), questions asked to parents or child (87.2%), and respiratory tests (59%).

In the visual assessment, orthodontists most often observed whether the child kept his/her lips sealed (97.2%) and his/her posture (80.0%). The remaining items observed were: presence of anterior open bite (67.2%), dark eye circles (63.6%), long face (63.6%), gingivitis in anterior maxillary teeth (50.9%), posterior cross bite (49%), and others (25.4%).

The questions often asked by orthodontists to parents or children were about the position of the lips, whether he/she sleeps or keeps his/her mouth open (90% and 86.3%). The remaining questions were about snoring (68.1%), drools on the pillow (66.3%), allergies (62.7%) whether the child becomes tired easily (59%), had a cold easily (24.5%) and others (15.4%).

The breathing tests most commonly applied by orthodontists to their pediatric patients were the lip seal test (75.4%), the mirror test (56.8%), and the water retention test (34.5%). Other tests cited by 5.4% of orthodontists were placement of cotton under the nostrils and the swallowing test.

In the second phase of the study, 687 children were examined and classified as nose breathers or mouth breathers using the proposed guidelines. The results included in Table 2 present the values for each group.

**Table 2.** Prevalence of age group, sex and main characteristics in mouthbreather and nose-breather groups.

Variable	Groups			
	Nose-breather		Mouth-breather	
	n	%	n	%
Age (years)				
6 - 7	102	19.6	32	19.2
8 - 9	218	41.9	77	46.1
10 - 11	183	35.2	48	28.7
12	17	3.3	10	6
Sex				
Male	236	45.4	90	53.9
Female	284	54.6	77	46.1
Lip seal				
Present	507	97.5*	107	64.1
Absent	13	2.5	60	35.9*
Facial type				
Mesofacial	404	77.7*	100	60.5
Dolichofacial	61	11.7	58	34.7*
Brachyfacial	55	10.6*	8	4.8
Open bite				
Absent	437	84.1*	128	76.6
Top	40	7.7	18	10.8
Present	43	8.2	21	12.6
Palate				
Normal	328	63.1*	77	46.1
Atresic	192	36.9	90	53.9*
Total	520	100	167	100

* statistically significant (p < 0.050).

**Figure 1.**
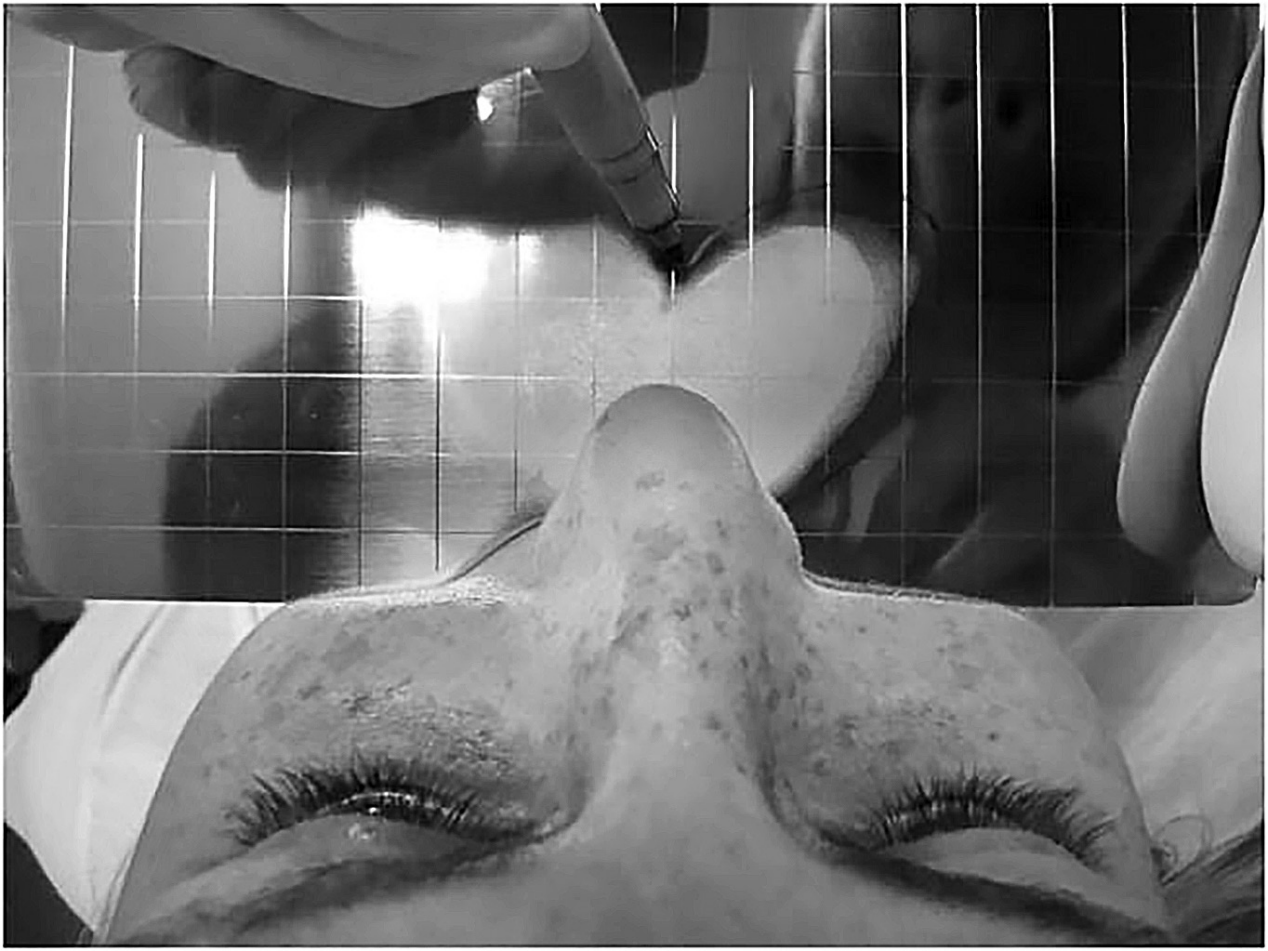
Marking the steam halo on the graded mirror test.

The absence of lip seal in 35.9% of mouth breathers and the presence of lip seal in 97.5% of nose breathers were both statistically significant. The predominant facial pattern in both groups was mesofacial; however, the presence of the dolichofacial pattern was high in the mouth-breather group (34.7%). Anterior open bite was found among 23.4% (top and present) of mouth breathers, a greater percentage in comparison to that found for the nose-breather group (15.8%). The presence of an atresic palate was significant in the mouth-breather group (53.9%).

Nearly one third of mouth breathers reported awareness of having problems during their sleep, such as sleeping with their mouth open or drooling on their pillow. Additionally, 18.6% reported awareness of snoring, whereas 34% felt daytime sleepiness. Regarding the questions of nasal and allergy problems, 31% of mouth breathers reported they usually have a runny or stuffy nose, whereas 30.5% sneezed frequently.

The results for the breathing tests carried out in all children are shown in Table 3.

**Table 3.** Prevalence of breathing tests in mouth-breather (MB) and nosebreather (NB) groups.

Variable	Groups				Total	
	NB		MB			
	n	%	n	%	n	%
Graded mirror test						
Halo greater than 30 mm	512	98.5*	143	85.6	655	95.3
Halo less than 30 mm	7	1.3	23	13.8*	30	4.4
Test not performed	1	0.2	1	0.6	2	0.3
Lip seal test						
3 minutes	510	98.1*	86	51.5	596	86.8
Less than 3 minutes	7	1.3	80	47.9*	87	12.7
Test not performed	3	0.6	1	0.6	4	0.5
Water retention test						
3 minutes	511	98.2*	90	53.9	601	87.5
Less than 3 minutes	5	1	76	45.5*	81	11.8
Test not performed	4	0.8	1	0.6	5	0.7
TOTAL	520	100	167	100	687	100

* statistically significant (p < 0.050).

Children classified as mouth breathers were those who had most severe obstructions (13.8%) and halos of steam measuring less than 30 mm. For those classified as nose breathers, this percentage was only 1.3%. Most children diagnosed as mouth breathers presented with bucconasal breathing (85.6%) and halos of steam greater than 30 mm.

For children classified as mouth breathers, the lip seal test and the water retention test were important in helping to diagnose whether MB was by habit or obstruction. Table 3 shows that half the group of mouth breathers were MB by habit. They could keep their lips sealed for up to 3 minutes (51.5% in the lip seal test and 53.9% in the water retention test).

## DISCUSSION

Although many articles describe the consequences of MB,^1^^,^ 2 ^,^ 6 ^,^ 11 few studies investigate the key parameters for clinical recognition of MB, especially in children.

The orthodontists interviewed for the present study consider the presence of sealed lips and the posture of the child as the most important aspects in determining whether a child is a mouth breather or nose breather. The presence of sealed lips in most children comprising the nose breather group and a statistically significant absence of sealed lips in the mouth-breather group were also found using the proposed guidelines. The agreement between the diagnostic impression of orthodontists and the clinical verification of the item "lack of lip seal" has also been shown in other studies.^6^^,^ 7 ^,^ 18

Felcar et al^7^ found absence of sealed lips in 58.8% of mouth breathers, and sagging and hypofunction of the orbicularis oris muscle were considered causes of lack of lip seal in 67% of mouth breathers.^18 ^Absence of sealed lips suggests the presence of vertical and sagittal facial discrepancies, inadequate lip length, increased lower facial height, abnormal breathing function, and altered lip tonicity. Increased lower facial height, a characteristic of the dolichofacial type, was also found in the present study. The presence of the dolichofacial type was statistically significant in the mouth-breather group.

The most prevalent malocclusions found in the mouth-breather group were atresic palate and anterior open bite. Several studies have confirmed the close relationship established between teeth, supporting tissues and the functional activity of the neuromuscular system.^6^^,^ 9 ^,^ 19 ^,^ 20 When abnormal pressure of muscles interferes in facial growth, it can determine the appearance of a malocclusion. The tongue can take a low and forward position, which is common in the presence of hypertrophic palatine tonsils as an attempt to increase posterior airway space and ease breathing. The low position of the tongue decreases internal pressure in the upper arch, increasing the external pressure of perioral muscles and causing an atresic palate.^6^^,^ 10 ^,^ 19 ^,^ 20 Because imbalance can cause anatomical and functional changes, proper balance between bones, muscles, and dental structures is essential.

In our study, most orthodontists asked whether the child had allergies. Regular allergic episodes are noteworthy and should be considered in the diagnosis. Temporary, but repeated obstruction of the upper airway can create the habit of breathing through the mouth. Most children with OSA have difficulty breathing through the nose. Allergic rhinitis is the most commonly cited disease, followed by hypertrophy of the tonsils and adenoids.^21^

By applying the guidelines to mouth breathers, we realized that nasal problems and sleep problems were the most relevant. Mouth breathers reported having nasal problems and awareness that they usually snore at night. Rates of snoring vary widely in the literature, depending on the age group studied or the questionnaire employed. Petry et al^12^ found a prevalence of 27.6% of habitual snoring, higher than what we found in the present study.

In assessing sleep-related problems, mouth breathers answered they wake up during the night, wake up with a dry mouth, and feel sleepy during the day. Popoaski et al^21^ reported a percentage of sleep problems of 37.7%, close to what was found in our study. These issues demonstrate the importance of asking patients about sleep and nasal problems during evaluation of mouth breathers.^22^

The mirror test and the water retention test are among the breathing tests most cited in the literature.^2^^,^ 7 ^,^ 15 ^,^ 23 ^-^ 26 However, these tests are not standardized and are described with little or divergent information in different publications.^2^^,^ 26 The lip seal test is not well described in the literature. In our study, breathing tests were hardly ever used by orthodontists, with no uniformity in the evaluation time for lip seal or water retention tests and lack of agreement on the manner of application of these tests. The lip seal test was the most frequently used, followed by the mirror test and the water retention test.

In order to standardize the breathing tests, we choose an evaluation time of 3 minutes.^26^ The choice of this longer period of time is justifiable because a mouth breather, even when the condition occurs due to obstruction, may breathe through the nose for a short period of time depending on the level of nasal obstruction. Breathing tests are useful to differential diagnosis, as they aid clinicians to decide on the most appropriate treatment modality.

The habit of breathing through the mouth, even without obstruction, alters the balance of facial muscles and causes the same facial skeletal changes that occur among MB due to obstruction.

The presence of MB by habit was also found in our study. Approximately half the group of mouth breathers managed to keep their lips sealed for up to 3 minutes during the lip seal test and the water retention test. Our guidelines provide orientation on how to restore the nasal breathing pattern of these children by performing the lip seal test every day at home for progressively longer periods each day.

When only a single breathing test is used, results are considered unreliable to determine whether the child is a mouth or nose breather. As guidance, this study suggests the use of at least two breathing tests together - the mirror test in combination with the water retention test or the lip seal test - so as to minimize errors in the recognition of a child's breathing pattern.

With a view to supplementing our diagnostic approach to MB and its immediate or delayed consequences that may lead to SDB, we observed that most orthodontists had some knowledge about SDB in adults. Currently, treatment of snoring and sleep apnea in adults has been widely included in several courses for dentists. However, childhood SDB presents characteristics that are quite different from SDB in adults.^3^^,^ 13 ^,^ 27 The presence of SDB, particularly snoring and OSA, is fairly significant among the pediatric population.^3^ UARS is highly prevalent during childhood, but it is little known by healthcare professionals.^11^^,^ 27 Questions about daytime sleepiness and difficulty concentrating at school should also be incorporated into the questionnaire.^13^

Attention deficit hyperactivity disorder (ADHD) is commonly found among MB children. When assessing children with ADHD and complaints about school underachievement, Costa et al^15^ found characteristics of snoring, nocturnal MB, rhinitis, tonsillitis, drool on the pillow, dark circles, and dry lips in more than half of their sample. Both ADHD and MB can trigger SDB, which, together with daytime sleepiness, directly interferes in school performance.^1^^,^ 15

The guidelines proposed herein should be used as reminders. Due to the importance of these disorders, we emphasize the need for early recognition of signs of SDB in children in order to minimize the occurrence of associated disorders in adulthood.

## CONCLUSIONS

To achieve clinical recognition of mouth breathing (MB), it is important for orthodontists to integrate results yielded by visual assessment, questions, and at least two types of breathing tests. It is essential to ask questions that help identify predisposing factors for sleep-disordered breathing in children. The proposed guidelines may favor the clinical recognition of MB in children, help differentiate between MB caused by habit or by obstruction, guide the clinician to choose the most appropriate treatment modality, and prevent adaptive facial changes that perpetuate the MB pattern.
